# Training-induced recovery of low-level vision followed by mid-level perceptual improvements in developmental object and face agnosia

**DOI:** 10.1111/desc.12178

**Published:** 2014-04-04

**Authors:** Maria Lev, Sharon Gilaie-Dotan, Dana Gotthilf-Nezri, Oren Yehezkel, Joseph L Brooks, Anat Perry, Shlomo Bentin, Yoram Bonneh, Uri Polat

**Affiliations:** 1 Faculty of Medicine, Goldschleger Eye Research Institute, Tel Aviv UniversityIsrael; 2 Institute of Cognitive Neuroscience, University College LondonUK; 3 School of Psychology, University of KentUK; 4 Department of Psychology, Hebrew University of JerusalemIsrael; 5 Center for Neural Computation, Hebrew University of JerusalemIsrael; 6 Department of Human Biology, University of HaifaIsrael

## Abstract

Long-term deprivation of normal visual inputs can cause perceptual impairments at various levels of visual function, from basic visual acuity deficits, through mid-level deficits such as contour integration and motion coherence, to high-level face and object agnosia. Yet it is unclear whether training during adulthood, at a post-developmental stage of the adult visual system, can overcome such developmental impairments. Here, we visually trained LG, a developmental object and face agnosic individual. Prior to training, at the age of 20, LG's basic and mid-level visual functions such as visual acuity, crowding effects, and contour integration were underdeveloped relative to normal adult vision, corresponding to or poorer than those of 5–6 year olds (Gilaie-Dotan, Perry, Bonneh, Malach & Bentin, 2009). Intensive visual training, based on lateral interactions, was applied for a period of 9 months. LG's directly trained but also untrained visual functions such as visual acuity, crowding, binocular stereopsis and also mid-level contour integration improved significantly and reached near-age-level performance, with long-term (over 4 years) persistence. Moreover, mid-level functions that were tested post-training were found to be normal in LG. Some possible subtle improvement was observed in LG's higher-order visual functions such as object recognition and part integration, while LG's face perception skills have not improved thus far. These results suggest that corrective training at a post-developmental stage, even in the adult visual system, can prove effective, and its enduring effects are the basis for a revival of a developmental cascade that can lead to reduced perceptual impairments.

## Introduction

Sensory experience dramatically shapes neural structures and functions during the early period of life, termed the ‘critical period’ (Horton & Hocking, 1997; Hubel & Wiesel, 1970). While different visual cortical structures and functions may develop at different rates (Atkinson, 2000; Levi & Carkeet, 1993), the general notion is that ‘basic’ functions typically develop earlier and more complex functions develop at a later stage, and recent studies show that visual functions processed at higher levels within the visual cortex have a later ‘critical period’ than functions processed at lower levels (Daw, 1998). Furthermore, functions involving higher cortical areas which reach maturity much later have a much shorter critical period (Ellemberg, Lewis, Maurer, Brar & Brent, 2002b). Consequently, a developmental cascade in time may be paralleled by a maturation hierarchy of cortical visual areas, where the maturation of each visual function or cortical visual area may rely on the maturation of the ones preceding it.

Amblyopia is a visual disorder manifested by monocular (anisometropia and strabismus) or binocular (binocular deprivation due to congenital cataract) reduction of visual acuity following abnormal binocular visual experience during the ‘critical period’ (Daw, 1998; Horton & Hocking, 1997; Hubel & Wiesel, 1970). It is characterized by several spatial vision abnormalities in the amblyopic eye (for reviews, see Ciuffreda, Levi & Selenow, 1991; Hess, Field & Watt, 1990; Levi, 1991; Levi & Carkeet, 1993) including reductions in visual acuity, contrast sensitivity, abnormal suppressive and facilitatory spatial interactions (Ellemberg *et al*., 2002b; Levi, Hariharan & Klein, 2002; Lewis, Ellemberg, Maurer, Wilkinson, Wilson, Dirks & Brent, 2002; Polat, Ma-Naim, Belkin & Sagi, 2004), as well as impaired contour detection (Hess, McIlhagga & Field, 1997; Kovacs, Polat, Pennefather, Chandna & Norcia, 2000) possibly due to the lack of collinear facilitation (Bonneh, Sagi & Polat, 2004; Ellemberg, Hess & Arsenault, 2002a; Polat, 2008; Polat *et al*., 2004).

While monocular amblyopia might affect mainly monocular processing (e.g. primary visual cortex, V1), with visual processing receiving already integrated binocular information relying on normal input through the fellow (sound) eye, binocular amblyopia, that is due to binocular congenital cataract, affects binocular processing at levels higher than V1 (Ellemberg *et al*., 2002b; Lewis *et al*., 2002). Nevertheless, even monocular amblyopia affects areas beyond V1 (Hess, Thompson, Gole & Mullen, 2010; Ho, Paul, Asirvatham, Cavanagh, Cline & Giaschi, 2006; Sharma, Levi & Klein, 2000). Thus, visual deprivation during the critical period affects visual processing at brain areas higher than V1, with binocular deprivation having a greater effect on visual processing than monocular deprivation (Ellemberg *et al*., 2002b).

An unusual case of abnormal visual development of a young male (LG) was recently described including neuroimaging examinations (Ariel & Sadeh, 1996; Gilaie-Dotan, Bentin, Harel, Rees & Saygin, 2011; Gilaie-Dotan *et al*., 2009). LG has developmental visual object and face agnosia resembling the ‘associative’ type (Biran & Coslett, 2003), but without other neurological disorders and no apparent cortical structural abnormality. He completed high school with high scores, and functions normally in all other aspects. fMRI and ERP examinations revealed that LG has significant ventral stream processing abnormalities (Gilaie-Dotan *et al*., 2009). His intermediate visual areas (V2 and V3) are abnormally deactivated in response to visual stimulation, and his higher-order category selective regions do not exhibit the expected object and face sensitivity. In contrast, his motion-sensitive area, MT+/V5, responds in a typical manner to visual motion, consistent with the finding that sensitivity to motion is typically normal in developmental prosopagnosia (Le Grand, Cooper, Mondloch, Lewis, Sagiv, de Gelder & Maurer, 2006).

At the onset of the current study, LG, aged 20, had a binocular reduction in visual acuity (VA) which did not result from a known optical deficiency and could not be corrected by spectacles. His visual acuity at that time was 0.5–0.6 LogMAR in each eye (visual acuity of 0.6 LogMAR (i.e. ‘20/80’) which is four times worse than the standard adult vision of 0 LogMAR (‘20/20’); LG's right–left eye Snellen equivalents: 6/19–2/24 m; 20/63/–20/80 ft) which could not be attributed to optical refractive error. Moreover, a significant part of the acuity deficit was due to visual crowding (∼0.3 log units, twice as poor as standard adult vision), as measured with crowded and uncrowded displays of tumbling E patterns (Bonneh *et al*., 2004). Two tests suggested abnormal early and mid-level integration mechanisms. First, in a lateral masking experiment (Polat & Sagi, 1993) LG exhibited no collinear facilitation, indicating impairment in local integration mechanisms. Second, in a contour-in-noise detection card test, testing mid-level contour integration mechanisms (Chandna, Pennefather, Kovacs & Norcia, 2001; Kovacs, Kozma, Feher & Benedek, 1999), his performance was significantly worse than normal adult vision, similar to that of 5–6 year olds (with a threshold spacing ratio of ∼1) (Kovacs *et al*., 1999). Contour integration is a fundamental mid-level stage in visual processing that involves cooperative interactions between local processing elements, with contributions to higher-level tasks such as perceptual grouping, image segmentation, and object recognition (Kovacs *et al*., 1999; Kovacs *et al*., 2000).

In the current study we sought to reduce LG's severe visual impairments, including mid-level visual functions and possibly also his object and face agnosia by means of visual training involving low-level visual mechanisms. We hypothesized that LG's mid-level and higher-level impairments stem from abnormal binocular input during development (with currently an unknown cause), a condition that resembles binocular amblyopia. Therefore, his condition prevented the typical developmental cascade, resulting in immature integration mechanisms and abnormal development of many visual functions.

Perceptual learning can improve visual functions in amblyopia (Levi & Li, 2009a, 2009b; Levi & Polat, 1996; R.W. Li, Ngo, Nguyen & Levi, 2011; Polat, 2009a, 2009b; Polat *et al*., 2004; Polat, Ma-Naim & Spierer, 2009; Polat & Sagi, 1994b). There is also an emerging effort to use video game playing as an additional training tool to enhance the participant's engagement, as for some populations the standard perceptual learning procedures might not be engaging enough. Although video gaming as a training tool is still in its infancy, it can also improve visual function in amblyopia (Jeon, Maurer & Lewis, 2012; R.W. Li, Klein & Levi, 2008; R.W. Li *et al*., 2011) and in normal vision (R. Li, Polat, Makous & Bavelier, 2009; R. Li, Polat, Scalzo & Bavelier, 2010). Here we further hypothesized that a corrective treatment for LG, based on standard perceptual learning procedures, even at the monocular level, would affect his binocular functions and might facilitate maturation of mid-level and perhaps even higher-level perceptual functions. To test this theory, we decided to train LG with the lateral masking paradigm (Polat *et al*., 2004). The lateral-masking paradigm is an established method of effective vision correction for various conditions such as amblyopia (Ellemberg *et al*., 2002a; Levi *et al*., 2002; Polat *et al*., 2004) and presbyopia (Polat, Schor, Tong, Zomet, Lev, Yehezkel, Sterkin & Levi, 2012), and has been extensively studied in psychophysical and physiological studies (e.g. Kapadia, Ito, Gilbert & Westheimer, 1995; Kasamatsu, Miller, Zhu, Chang & Ishida, 2010; Kasamatsu, Polat, Pettet & Norcia, 2001; Lev & Polat, 2011; Mizobe, Polat, Kasamatsu & Norcia, 1996; Solomon & Morgan, 2000; Sterkin, Yehezkel, Bonneh, Norcia & Polat, 2008; Woods, Nugent & Peli, 2002). Therefore, we employed lateral-masking treatment of amblyopia (Polat *et al*., 2004) especially tailored for LG, by training him twice a week for a few months, monocularly and binocularly. The results show a marked improvement in visual acuity, crowding, and mid-level functions including contour integration, with possible subtle improvement in object recognition and part integration, but not in face perception.

## Methods

### Case description

LG was 20 years old at the beginning of this study (before starting the training). He is a right-handed male and has developmental visual agnosia and prosopagnosia. LG's perceptual impairments were already evident in early childhood (Ariel & Sadeh, 1996). A recent study examined his low-level visual functions and high-level visual performance, including reading, object, and face recognition (Gilaie-Dotan *et al*., 2009). A brief description of his low-level vision, as described in that study, is described within the Results section (pre-training performance). That study also reported fMRI and ERP neuroimaging investigations of LG's visual system, and a high-resolution structural MRI scan of his brain, which was examined by a neuro-radiologist who was blind to LG's condition and found no evidence of structural abnormality. LG has always functioned as a fully independent person and successfully finished high school. He is now a university student, and works, reads, and travels on his own. The current study started about a year after that study took place (Gilaie-Dotan *et al*., 2009).

Several visual functions including LG's basic, mid- and high-level vision were tested before the training began (‘pre-training’), during, and after training (‘post-training’). In addition, we administered to LG an additional set of mid-level vision tests only post-training to assess more thoroghly his mid-level visual functioning.

### Low-level vision

**Figure 1:**
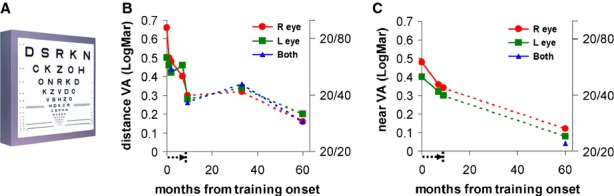
LG's visual acuity (VA) improvements during training are long lasting. (A) Illustration of a visual acuity ETDRS chart. (B) LG's VA for distance (3 meters); (C) LG's VA for near (40 cm). The x-axes show the time since the onset of training, the arrow below the x-axes (0–9 months) indicates the duration and cessation of the training period, the y-axes denote visual acuity in LogMAR (on the right) or Snellen units (on the left), lower values indicate better VA. The red, green, and blue plots show the results for right (R), left (L), and both eyes, respectively. Both distance and near VA improved significantly and are enduring for more than 4 years post-training.

*Visual acuity* (VA) was measured according to the best visual correction at a distance of 3 m (distance VA) or 40 cm (near VA), using a modified Bailey–Lovie (LogMAR) chart (ETDRS, see Figure 1A). We report LogMAR units as well as their Snellen equivalents.

**Figure 2:**
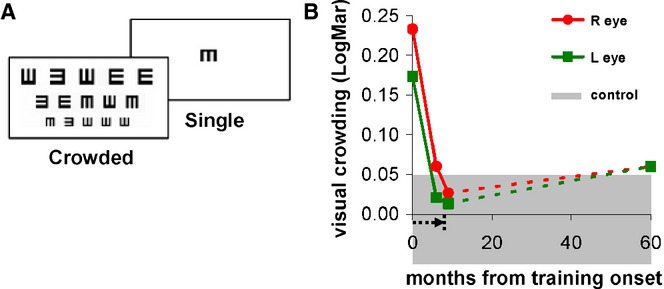
Visual crowding improvements during and following training. (A) Stimuli examples for measuring crowding effects and visual acuity by the ‘Tumbling-E patterns test’ (Bonneh et al., 2004), which is a LogMAR chart equivalent. The minimal size for central pattern identification is measured under single (on left) and multiple (crowded, on right) pattern conditions to determine visual acuity; crowding effects are estimated from the acuity difference between the crowded and single conditions, smaller crowding effects indicate better vision. The x-axis shows the time since the onset of training (arrow below the x-axis indicates the duration of the training), and the y-axis denotes the crowding effects in LogMAR units. Visual crowding was measured pre-training (at x-axis = 0), during and at the end of the training (x-axis = 9), and more than 4 years post-training (x = 60 months). The red and green indicate right (R) and left (L) eyes, respectively. The gray shaded zone denotes normal crowding effects of the normal adult population (0 ± 0.05 (SEM)).

The *crowding effect* was measured in an identical way to that described previously (Bonneh *et al*., 2004). In short, VA was measured by a ‘Tumbling-E patterns test’ which is a LogMAR chart equivalent, monitor-based paradigm (see Figure 2A). The stimuli correspond to a subset of the LogMAR chart. Pattern size corresponding to 6/6 (20/20) on a Snellen chart is equivalent to 0.0 on a LogMAR chart. Three rows of five black E-patterns each, on a white background, each pattern facing one of four directions (crowded pattern). The task was to determine the direction of the central E. Auditory feedback was provided with different tones for correct and incorrect responses. A staircase procedure with pattern size and spacing modified by 0.1 log units in each step was used to determine the size for 50% accuracy (chance is 25%). The performance on a central E, presented alone (single pattern), was measured separately. The crowding effect was then calculated by *subtracting the crowded from the single* pattern results (difference on a log scale), in other words, normalizing the crowded condition according to the acuity of a single pattern.

**Figure 3:**
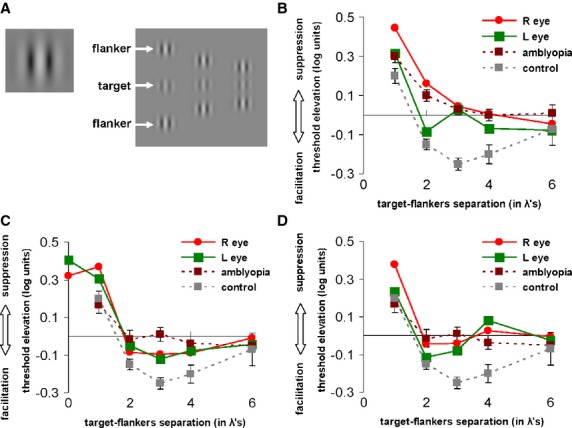
Lateral interactions before and after LG's training. (A) Collinear lateral interactions stimuli examples, in which contrast detection threshold of a single flashed Gabor patch at fixation (on left) is measured against detection threshold of displays with lateral high contrast collinear flankers (appearing above and below target) at various distances (on right, three columnar examples of different distances). (B–D) The x-axes denote target-flankers separation in wavelengths (λ), y-axes denote the contrast detection threshold difference (single target vs. with flankers) in log units. (B) Before training LG's lateral interactions were dominated by suppression, especially for 2–3 λ (LG's right (R) and left (L) eyes denoted in red and green, respectively), similar to those found in typical adult amblyopia before training (dashed brick line), and not showing the typical facilitation effect found in normal adults (dashed gray line). (C) After 9 months of training, LG's lateral interactions for λ ≥ 2 (typically showing facilitation in healthy adults controls) showed small but clear facilitation effects, similar to amblyopes following training (dashed brick line), and these effects in LG endured over 4 years post-training (D) for 2–3 λ. The control data presented in panels B, C, and D are the same for all these panels; lateral interactions of typical amblyopes are presented in B, and interactions of amblyopes following training are presented in panels C and D. Error bars indicate SEM.

*Lateral interactions* were measured in the current study following procedures described earlier (Polat *et al*., 2012; Sterkin, Yehezkel & Polat, 2011). In short, stimuli consisted of Gaussian-modulated grating signals (termed a ‘Gabor patch’) with spatial frequencies of 3, 6, or 9 cycles per degree modulated from a background luminance of 40 cd/m^2^ (Figure 3A), with the standard deviation of the Gabor patch equal to the wavelength (σ = λ). The contrast detection threshold was measured by a two-interval forced choice procedure, in which the target, which was presented for a duration of 60 to 120 ms, depending on the contrast threshold, appeared randomly in one of two intervals separated by 800 ms. A visible white fixation circle indicated the location of the target before each trial began. LG, control, and amblyopic participants started each trial by pressing a button and auditory feedback was given following wrong answers only. A 3:1 staircase method was used to determine the contrast threshold level of the target at 79% correct performance (Levitt, 1971) with steps of 0.1 log units; a test block was terminated after eight reversals of the staircase procedure, and the geometric mean of the last six reversal values was used to estimate the contrast threshold. Lateral masking was measured similarly, with additional flankers at different distances (2, 3, 4, and 6 wavelengths from the target), which appeared in both of the two interval forced choice displays. Backward masking was measured by introducing a varied temporal displacement between the onsets of the target and the flankers (Polat *et al*., 2012; Sterkin *et al*., 2011).

*Stereo acuity* was measured using a Randot Stereo test, which tests the ability to identify geometric forms from a random dot background using polarized glasses. Charts with a stimulus disparity of 20–600 seconds of arc were used at a distance of 40 cm from the participant's eyes to determine the minimal disparity needed to extract depth information.

### Mid-level vision

**Figure 4:**
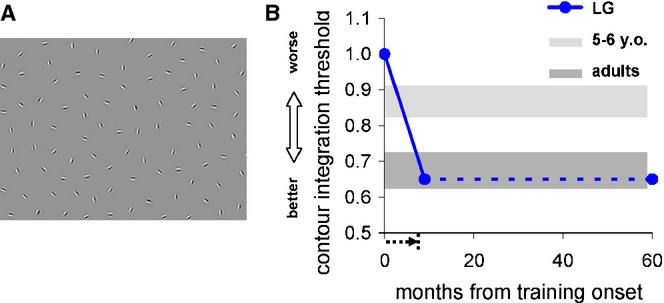
LG's contour integration threshold improved to age-level performance. (A) Illustration of contour detection in noise stimulus, where a contour made of Gabor patches (circular shape) embedded in randomly oriented patches was measured as a function of background element density to target spacing ratio. See Methods for more details. (B) LG's performance pre-training to more than 4 years post-training. The x-axis shows the time since the onset of training (arrow below x-axis indicates the training duration), and the y-axis denotes the contour integration thresholds. Light and dark gray lines indicate performance of children aged 5–6 years, and normal adults. Error bars indicate SEM.

*Contour detection threshold* was measured using charts consisting of a smoothly aligned, closed path of Gabor patches embedded in a randomly oriented array of Gabor patches of identical spatial frequency and contrast (Figure 4A; Kovacs *et al*., 2000). The angular difference between adjacent contour segments was assigned within a range of 0 to 30°. Each card was presented and correct or incorrect answers were recorded. The threshold was defined as the average spacing between background elements relative to the average spacing between the contour elements that allowed successful contour detection. This paradigm was identical to that described earlier (Kovacs *et al*., 2000).

*Motion coherence threshold* was measured in the current study following procedures described earlier (Gilaie-Dotan, Kanai, Bahrami, Rees & Saygin, 2013a; Gilaie-Dotan, Saygin, Lorenzi, Egan, Rees & Behrmann, 2013b). In short, in a two-interval forced choice experiment, each trial consisted of two intervals, each displaying a disc of moving dots, and participants had to detect the disc that consisted of coherently moving dots (target) while the other disc (distracter) consisted of white noise only. The amount of coherent motion in the target disc was adjusted in an adaptive manner in order to estimate individual coherence thresholds that allow performance accuracy of 75%.

*Perceptual organization mechanisms* were measured with the Leuven Perceptual Organization Screening Test (L-POST, see http://gestaltrevision.be/tests; Crawford, Garthwaite & Slick, 2009; Torfs, Vancleef, Lafosse, Wagemans & de-Wit, in press) which is a new diagnostic tool for detecting perceptual mid level impairments. LG's performance was compared to norms of more than 1500 individuals. Percentile scores of the test are based on established neuropsychological procedures (Crawford *et al*., 2009).

### High-level vision: object recognition and part integration

LG's object recognition and part integration were measured using different tests:

The Birmingham Object Recognition Battery Test no. 6 (Riddoch & Humphreys, 1993) measures the ability to identify letters, geometric forms, and line drawings, which are shown either separately, near each other or overlapping. One important measure is the ratio between the reaction times (RTs) for naming pairs or triplets of objects when they are not overlapping versus overlapping. Normal object recognition is determined by a ratio of or approaching 1:1.

The Hooper Visual Organization Test (Hooper, 1983) measures an individual's ability to spatially organize visual stimulus parts. This task is considered particularly sensitive to neurological impairments such as visual agnosia. The test consists of 30 line drawings, each showing a common object or animal – such as a ball or a fish – that has been cut into several pieces. The pieces are scattered on the page like parts of a puzzle. The participant's task is to say what the object would be if the pieces were put back together correctly.

The Visual Object and Space Perception Battery (VOSP) is a set of tests that examine object and space perception relative to norms of brain damaged patients, including object perception screening, incomplete letters, silhouettes, object decision, and progressive silhouettes subtests, as well as a few space perception subtests, from which LG was only tested on the cube analysis.

The completion experiment measures individual ability to name line drawings of animals either from intact (‘whole’ condition) or from partly hidden displays when a grid of vertical stripes is superimposed on them (‘grid’ condition, see Figure 6C). The experiment is also described elsewhere (Gilaie-Dotan *et al*., 2009; Lerner, Hendler, Ben-Bashat, Harel & Malach, 2001). Performance was measured as naming accuracy either at the basic (e.g. ‘cat’) or at the subordinate level (e.g. ‘lion’, ‘cheetah’). Each stimulus was presented for 500 ms, followed by a 1500 ms (controls) or 3000 ms (LG) blank screen during which the overt naming of the image has been recorded. LG was tested pre-training as described previously (Gilaie-Dotan *et al*., 2009). Post-training, LG was tested more than 4 years after training ended. In that testing session, he was tested with the original line drawings, but also with new and unfamiliar line drawings of animals of similar difficulty, to account for possible familiarity influences on his performance.

### High-level vision: face recognition

LG's face recognition was assessed by the the Benton Face Recognition Test (BFRT; Benton, Sivan, Hamsher, Varney & Spreen, 1994) at the same time that we assessed his object recognition skills (more than 2 years post-training). In each trial item, LG was presented with a target face placed above six test faces, and was asked to indicate which of the six images below matched the target face. There are no time limits for any item. Male and female face images were used in this test; they were cropped so that no clothing and little hair were visible. In the first six trials, only one of the six test faces matched the target face, and the target image and the test image were identical. In the next 16 trials, however, three of the test faces matched the target face, and the poses of the test images or their lighting conditions were different from those of the target image. The task was to detect all the test faces that are the same as the target, with a point for each correct detection.

### The training procedure

**Figure 5:**
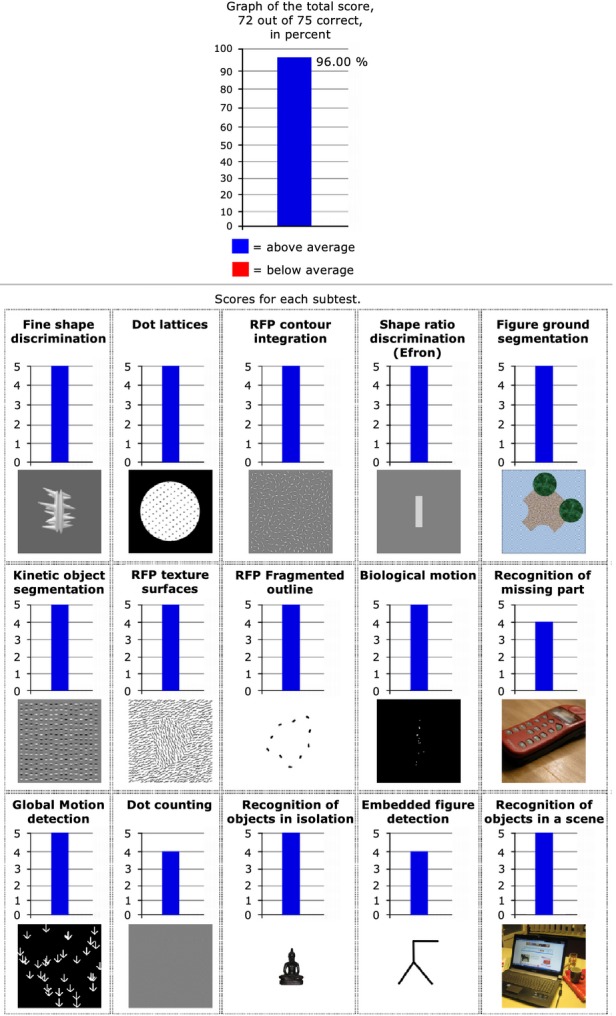
LG's normal performance on a wide range of mid-level visual functions. Top: LG's overall performance across all tests from the Leuven Perceptual Organization Screening Test (L-POST, see Methods), below: LG's scores for the specific sub-tests. LG scored above average on all subtests (in blue). Norms are based on performance of more than 1500 individuals. Permission to duplicate L-POST output results obtained from Johan Wagemans.

The perceptual learning procedure that we applied for training LG's vision used princples similar to previous training studies (Polat *et al*., 2004; Polat & Sagi, 1994b; Polat *et al*., 2012). These studies indicate that the training resulted in improved spatial and temporal neural processing. In each session, LG trained on tasks that included (a) contrast detection of a Gabor target, (b) contrast detection of a target arranged between two other Gabor flankers arranged in collinear configuration (also termed ‘lateral masking’), (c) lateral masking followed by delayed identical flankers without a target (termed backward masking), as described previously (Polat *et al*., 2012; Sterkin *et al*., 2011). The training covered a range of spatial frequencies (3–12 cycles per degree) and orientations (0, 45, 90, and 135 degree angles); the inter-stimulus interval between the target and the backward masking stimulus was reduced (from 180 ms to 60 ms) according to the progress of the training. LG trained from a distance of 150 cm either monocularly (either right or left eye in separate conditions), or binoculaly (both eyes open). Auditory feedback was provided with different tones for correct and incorrect responses. The training, which was performed in a darkened room, took about 30 min per session, and took place at least three times a week for a continuous period of approximately 9 months. Following this period, LG stopped training for a period of 4 years. After 4 years off training (i.e. approx. 57 months from training onset), LG decided he wanted to return to training for a few sessions. So the measurements from the ‘60 months from training onset’ time point (in Figures 1, 2, 3D, 4, and 5) were taken during that period, after LG had returned to training for a few sessions following the 4 years off training.

## Results

### Low-level vision

#### Visual acuity

Before training, LG's distance visual acuity (VA) was abnormal: 0.66 LogMAR in his right eye (6/30− m; 20/200− ft) and 0.5 LogMAR in his left eye (6/18 m; 20/63 ft) (see Figure 1B, left panel). This level of vision resembled a condition of binocular amblyopia (Ellemberg *et al*., 2002b; Lewis *et al*., 2002). After 9 months of training, LG's distance VA improved by more than a factor of 2 for the right eye (from 0.66 to 0.3 LogMAR units, ≈ 109%; from 6/30− m; 20/200− ft to 6/12 m; 20/40 ft), and by 66% for the left eye (from 0.5 to 0.28 LogMAR units; from 6/18 m; 20/63 ft to 6/12 + m; 20/40 + ft), as presented in Figure 1B. The improvement in LG's right eye was higher (109%) than the average reported improvement (about 80%) that follows a similar perceptual learning technique for amblyopia and presbyopia (Polat, 2008, 2009a; Polat *et al*., 2004; Polat *et al*., 2012).

Before training, LG's subjective refraction showed no improvement on the visual chart for any myopic prescription, and thus no glasses were recommended at that stage. After training, however, his objective refraction (retinoscopy) showed a small myopic optical error, and therefore the best optical correction (−0.5 diopter in each eye) that met the objective prescription was prescribed. Consequently, LG was able to discriminate better between the letters on the distance visual chart using the small myopic correction than without the optical correction (the post-test of distance VA performed without the new optical correction). His level of binocular distance VA after the training was below 0.3 LogMAR (6/12 m; 20/40 ft) within the range that legally qualifies him for a driving licence.

LG's near VA improved from 0.48 to 0.34 (38%, 6/19 + to 6/12− m; 20/63 + to 20/40− ft) and from 0.4 to 0.3 LogMAR units (26%; 6/15 to 6/12 m or 20/50 to 20/40 ft) for the right and left eyes, respectively (see Figure 1C, right panel). This VA enabled him to read a letter size of 6.3 points, slightly larger than the common newspaper letter size. LG was tested 24 months after his training ended (33 months after training onset) and his distance VA was found to be stable (right eye: 0.32, left eye: 0.34, in LogMAR units, see Figure 1B). Furthermore, 60 months after training onset (51 months from the end of training), LG's near and distance VA have improved even further (see Figure 1B and C).

#### Visual crowding

The normal range of the crowding effect in control adult subjects tested with this technique is around zero (0 ± 0.05 LogMAR; mean ± SEM; Bonneh *et al*., 2004; Bonneh, Sagi & Polat, 2007; Doron & Polat, 2011). Before training, LG's crowding effects were much higher (right eye, 0.23; left eye 0.17) and were reminiscent of the crowding effects pronounced in strabismic amblyopia (Bonneh *et al*., 2004, 2007; Jeon, Hamid, Maurer & Lewis, 2010; Levi, 2008; Pelli & Tillman, 2008), and in young children up to the age of 7 (Doron & Polat, 2011). After training, however, LG's crowding effects were reduced, reaching normal adult level, as shown in Figure 2B. LG's crowding improvements were maintained close to adult normal range even 60 months after training onset. For comparison, crowding effects in 10 normally sighted adults were not significantly different on a test–retest basis (*t*(8) = 0.11, *p* > .9, paired *t*-test).

#### Lateral interactions

Prior to training, LG's lateral interactions (Figure 3A) were measured for a spatial frequency of 3 cycles per degree. In normal vision, facilitation is found for target-flankers separation of 2–6 wavelengths (λ), being maximal at 3λ and decreasing with increased target-flankers separations (Polat & Sagi, 1993, 1994a, 1994b), as shown in Figure 3B for controls. The function of lateral interactions in LG before training was different with very minimal facilitation in his left eye and dominated by suppressive effects for his right eye (Figure 3B). These suppressive effects in his right eye resembled strabismic amblyopia, and his left eye, with less severe suppression, resembled anisometropic amblypoia (Bonneh *et al*., 2004, 2007; Ellemberg *et al*., 2002a; Levi *et al*., 2002; Polat, 2008; Polat, Bonneh, Ma-Naim, Belkin & Sagi, 2005; Polat *et al*., 2004) and supports our hypothesis that LG's vision before training resembled effects found in amblyopia. After training (Figure 3C), suppression-wise, LG's suppression was reduced remarkably by about 0.15–0.2 log units in his right eye (41–58%), while facilitation-wise, both eyes reached facilitation levels that resembled amblyopes' facilitation post–training (see Figure 3C; Polat *et al*., 2004). The effect of improvement was even more impressive because at the beginning of the training LG was able to practice only on Gabor patches of 3 cycles per degree (the highest spatial frequency he could detect), but after 9 months of training he was able to detect and practice on Gabor patches of 9 cycles per degree. Furthermore, the changes in LG's facilitation endured, so that even more than 4 years post-training, both of his eyes still showed facilitation effects for target-flankers separation of 2–3λ (Figure 3D). This pattern of improvement with training is similar to that found in amblyopia following training (Figure 3C), leading to improvements in other visual functions such as VA, contrast sensitivity function, and binocular vision (Polat *et al*., 2004).

#### Stereopsis

LG's stereo acuity, measured with the RanDot test (RanDot, Stereo Optical Co., Inc.), improved from a threshold of 30 arcsec before training (within the normal range of adults) to 20 arcsec 6 months after training onset, which is the finest stereo sensitivity that can be measured with this test, with less than half of the adult population reaching this sensitivity level (Birch, Williams, Drover, Fu, Cheng, Northstone, Courage & Adams, 2008). This improvement does not seem to stem from test–retest effects, as the RanDot test shows high test–retest reliability (Birch *et al*., 2008) and our control data also supports this. From 10 age-matched controls that were tested twice with RanDot, as LG was, four had stereo acuity worse than 20 arcsec (i.e. 25 arcsec), and only one of these improved in the retest session, while the other three remained at 25 arcsec sensitivity.

### Mid-level vision

#### Contour integration

LG's contour integration threshold ratio (background element density to target spacing ratio, see Figure 4A) before training was ∼1, impaired relative to normal adult vision (see Figure 4B), and corresponding to that of 5–6 year olds when using a similar technique (see Figure 4B). After training, however, LG's contour integration threshold reached 0.65 (see Figure 4B), which is within the range of normal adults, typically reaching maturity at the age of 9 (Doron & Polat, 2011; Kovacs *et al*., 2000). This threshold was maintained for more than 4 years after the training ended (Figure 4B).

#### Motion coherence

Motion coherence threshold, which is considered a mid-level vision process (Le Grand *et al*., 2006), was assessed in LG only 3 years post-training. His coherence threshold reached 18.39%, not significantly different from the threshold of 32 age-matched controls with normal or corrected to normal vision aged 24.3 ± 5 (*SD*) years (controls' mean coherence threshold 14.6% ± 4.1% (*SD*) (Gilaie-Dotan *et al*., 2013a), LG vs. controls: *t*(31) = 0.909, *p* > .37 (Crawford & Howell, 1998)).

#### Perceptual organization

To assess a wide range of mid-level visual abilities following training in LG and to compare his mid-level visual performance to normative data, we also assessed LG's mid-level vision using the Leuven Perceptual Organization Screening Test (L-POST, see Methods) which is based on normative data of more than 1500 individuals. This test assesses functions such as fine shape discrimination, contour integration (similar to our contour integration assessment), global motion detection (similar to our motion coherence assessment), recognition of missing parts, embedded figure detection, figure–ground segmentation from static cues, kinetic object segmentation, and more. LG's performance was within the normal range for all the subtests, as can be seen in Figure 5, and this is consistent with results we obtained while assessing LG on some of these visual functions independently (e.g. motion coherence and contour integration in the current study, biological motion (Gilaie-Dotan *et al*., 2011), and figure–ground segmentation based on dynamic local cues (Brooks, Gilaie-Dotan, Rees, Bentin & Driver, 2012).

### High-level vision

#### Object recognition and part integration

**Figure 6:**
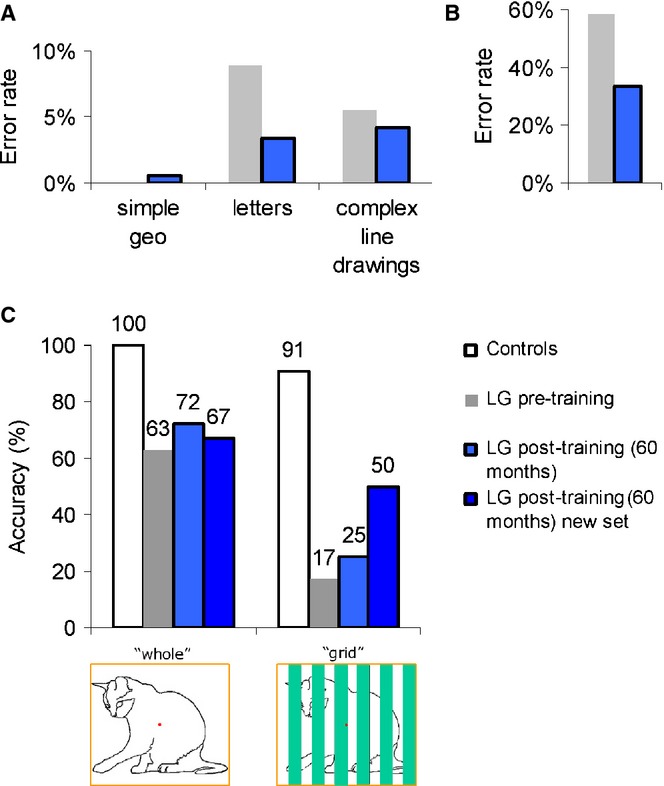
The error rates for object recognition and part integration before (in gray) and 4 years after the training ended (post-training, in blue). (A) LG's error rates in the Birmingham Object Recognition Battery overlapping figures task (test 6) were reduced following training only for letters (NB: LG's error rate on the pretest for simple geometric patterns was 0%). (B) LG's Hooper visual organization error rates dropped to nearly half following training (from 58% pre-training to 33% post-training). (C) LG's naming accuracy for intact or occluded line drawings of animals. His performance on the occluded animals might indicate mild improvement.

LG's performance on the overlapping figures test of the Birmingham Object Recognition Battery was better with simple geometrical shapes than with letters or more complex line drawings, both pre- and post-training. His difficulty with letters and complex line drawings was reflected both in his errors (see Figure 6A) and in his RT ratios. Pre-training, LG made no mistakes in the 180 geometrical shapes, but he made 16 errors in the overlapping pairs and triplets of letters (out of 180 letters all together), and four mistakes in the paired-overlapping line drawings (out of 36 pairs; 72 drawings all together). To minimize familiarity effects, we tested LG 4 years post-training on these tests (more than 4.5 years between testing sessions). Post-training, LG made six errors in the paired and triplet-overlapping letters section (mostly between similar letters, e.g. I and J); three mistakes in the paired-overlapping line drawings, and one mistake in the geometrical shapes. There was also an improvement in the average RTs under most conditions, as evidenced by shorter post-training reaction times. Another aspect relevant to the norms of the overlapping figures test from the Birmingham Object Recognition Battery is the RT ratio (between RTs of isolated stimuli and RTs of overlapping stimuli). Pre-training, LG's RT ratios were as follows: Paired letters 1:2.8, Tripled letters: 1:2.1; Paired line drawings: 1:2.7; Paired geometrical shapes: 1:1.2, Tripled geometrical shapes: 1:1.6, whereas post-training, LG's ratios were as follows: Paired letters 1:2.1, Tripled letters: 1:2.3; Paired line drawings: 1:2.3; Paired geometrical shapes: 1:1.2, Tripled geometrical shapes: 1:1.5. For the simple geometric shapes and the complex line drawings the pre-training and post-training error rates were mostly similar (see Figure 6A). However, even though LG's performance was still below the norm of the RT ratios (i.e. close to a ratio of 1:1), an improvement was seen in the number of errors in the letter section (see Figure 5A), in the absolute RTs under most of the conditions, and some improvement in the RT ratios was evident, especially for paired letters.

LG's pre-training score on the Hooper Visual Organization Test was 12.5/30, defined as a ‘very high probability of impairment’, whereas his post-training score was 20/30, defined as a ‘moderate probability of impairment’ (see Figure 6B).

LG was tested on the Visual Object and Space Perception Battery more than 4 years post-training. LG passed all the object perception subtests (when compared to norms of aged < 50 y.o.) except the object decision test (he scored 13 points and the pass cutoff is at 15), placing him above the 5% cutoff scores of these tests. He also passed the Cube analysis space perception subtest.

LG's pre-training and post-training performance in the completion experiment is shown in Figure 6C. His animal recognition ability was impaired even for images presented in the intact view condition (‘whole’, Figure 6C), before and after training, indicating on his difficulty in recognizing animals from such feeble stimuli. No apparent improvement was evident post-training for these intact ‘whole’ images. In the ‘grid’ condition, however, when LG had to overcome the occluding bars to recognize the animals, while before training his recognition was at 17%, post-training he successfully recognized 25% of the animals from the previously viewed set of pictures, and 50% from the newer set of line drawings that were unfamiliar to LG. We do not anticipate that the newer line drawings would be easier to recognize since in the intact condition LG did not perform better in recognizing them.

Thus, although LG is still considered impaired in object recognition, the tests we administered indicate that his object recognition abilities might have moderately improved following training.

#### Face recognition

In the Benton Face Recognition Test, LG scored 33/54 pre-training and 32/54 post-training, scores categorized as ‘severe impairment’ in face recognition. Thus, there was no indication of improvement in his face recognition abilities.

## Discussion

In this study we applied a dedicated visual corrective training programme to a 20-year-old man following abnormal visual developmental trajectory. Before training, LG's basic visual functions such as visual acuity, crowding effects, and contour integration abilities were underdeveloped relative to normal adult vision, and similar to those of 5–6 year olds or younger children, resembling amblyopic vision (Gilaie-Dotan *et al*., 2009). LG also suffered from higher-level visual perceptual impairments in face and object recognition (prosopagnosia and object agnosia). We monitored LG's visual improvements during and after training, as well as 4 years post-training. Importantly, the training significantly improved many of LG's basic visual functions (including visual acuity, contour integration, and crowding effects), approaching close to age-level performance, and this improvement was found to be stable and sustained even 4 years post-training. A mild improvement was also observed in LG's part integration and object recognition, although objective and subjective measurements indicate that LG still has object and face recognition impairments (but see Duchaine & Nakayama, 2006).

When LG was 20 years old, upon commencing the training, his visual system's estimated biological age was assessed as being around 5–6 years old (Gilaie-Dotan *et al*., 2009) or younger (cf. visual acuity performance). This estimate was based on the observation that in all the parameters that were tested (visual acuity, crowding, lateral interactions, and contour integration), LG's visual performance before training in either eye was similar to that of an amblyopic eye (Bonneh *et al*., 2004, 2007; Levi *et al*., 2002; Polat *et al*., 2004; Wong, Levi & McGraw, 2005) and corresponded to the developmental level of children (Doron & Polat, 2011). Additional factors supported this estimate. First, before the training, LG's high degree of suppression in measures of lateral interactions, which was paralleled by an increased effect of crowding and a deficit in contour integration, was underdeveloped relative to normal adult vision and similar to that found in 5–6 year olds (Doron, Meshulam, Mandel, Rosner, Belkin & Polat, 2007). Second, LG's perceptive field size (a psychophysical measurement of local integration fields) before the training was similar to the size of the perceptive field in the periphery, resembling effects observed in children when their fovea is not fully developed (Provis, Diaz & Dreher, 1998), as well as in amblyopia (Lev & Polat, 2011; Levi, Klein & Aitsebaomo, 1985). It has recently been shown that the human perceptive field in the fovea can be estimated from the collinear interaction function, at the cross-over point between suppression and facilitation (Lev & Polat, 2011, 2012). Based on that, our estimate of the size of LG's perceptive field in the fovea before training was 4λ for the right eye and 3λ for the left eye, an estimate that was larger than normal by 100% and 50% for the right and left eyes, respectively, and resembled the size of the perceptive field in the periphery. Taken together, we suggest that LG's vision prior to training was characterized by immature and undeveloped fovea relative to adult normal vision, similar to adult peripheral vision and in some aspects similar to vision of children about 5–6 years old.

Our results indicate that non-invasive intervention in the form of corrective visual training can be very effective in partially overcoming abnormal development of basic visual functions, even when such an intervention is applied years after the critical period. However, how influential can such a recovery of basic visual functions be to mid-level functions as contour integration and higher visual perceptual functions such as face and object recognition? Since normal development of perceptual functions probably relies on normal visual inputs, which were not present in LG, his perceptual functions did not develop normally. Could an improvement of perceptual functions take place after the recovery of basic visual functions due to corrected inputs that enter the visual system? Some support is evident from studies that tested individuals with congenital cataract (Maurer, Lewis & Mondloch, 2005; Maurer, Mondloch & Lewis, 2007; Ostrovsky, Andalman & Sinha, 2006). These studies suggest that visual functions and, consequently, perceptual functions were impaired in adults after removing the obstacles that prevented normal visual input during the normal developmental period. However, some perceptual functions recovered a few years after the brain received normal visual input. With respect to LG, we believe he suffered from unknown and unusual binocular deprivation of normal visual input during his childhood. This obstacle was a dominant cause that arrested the development of normal visual functions and hence some of his mid- and high-level perceptual visual functions. Less than a year after training onset, significant improvements in mid-level functions such as contour integration were already evident, and these were sustained for a long period. Additional mid-level functions tested years post-training appear normal. A possible mild improvement in LG's high-level shape recognition might be observed following training, when examining his possibly improved Hooper and Birmingham Object Recognition Battery overlapping figures test scores for letters, and possibly improvements in overcoming occlusion, yet the significance of these changes cannot be determined. LG also reported an improvement in his day-to-day life activities and felt more confident, partly due to his spectacles. However, LG still has significant impairments in object recognition and face recognition. This is evident from his significantly long reaction times in identifying objects, and his impaired face recognition. Furthermore, a recent study that was done 2 years post-training found that some aspects of LG's ability to perform figure–ground segmentation, which is assumed to support object recognition processes, are still impaired. For instance, although he can clearly perform figure–ground organization based on local motion cues, he does not appear to use contextual/non-local motion information to determine figure–ground organization (Brooks *et al*., 2012), something that control participants use very reliably (Brooks & Driver, 2010). LG also indicated that despite feeling more confident with his vision, he still does not feel that there is a significant improvement in his recognition skills. Thus, it remains to be seen whether there will be further improvement in his high-level perceptual functions of object and face recognition in years to come.

While LG is a patient individual, there are others who find it difficult to follow perceptual learning training procedures (e.g. children, ADHDs, and some clinical populations). Therefore, there is an ongoing effort to design and develop more engaging training protocols. As part of this effort, video gaming effectiveness has been studied, based on the idea that such games train the player on fine and rapid visual discrimination under conditions of high arousal and attentional engagement. The results of these studies show significant improvements in visual functions in normal vision (R. Li *et al*., 2009; R. Li *et al*., 2010) and in amblyopia (Jeon *et al*., 2012; R.W. Li *et al*., 2008; R.W. Li *et al*., 2011), although not by all types of video games (R. Li *et al*., 2009; R. Li *et al*., 2010). Since video gaming as a treatment tool is still in its infancy, more studies are required to establish this method and characterize the games and procedures that are most effective for improving visual functions.

Recent studies by Grill-Spector and colleagues that investigated the developmental trajectory of face sensitivity with respect to face perception in the visual cortex (Golarai, Ghahremani, Whitfield-Gabrieli, Reiss, Eberhardt, Gabrieli & Grill-Spector, 2007; Golarai, Liberman, Yoon & Grill-Spector, 2010; Grill-Spector, Golarai & Gabrieli, 2008) found that in normally developed individuals it takes more than a decade from the age of 7 for face-sensitivity in visual cortex to mature (Golarai *et al*., 2007), and that face-selective cortex in adults is significantly different from that of adolescents (Golarai *et al*., 2007). If indeed LG's visual system was underdeveloped relative to adult normal vision before training, some functions being at the biological age of 5–6 year olds, then a decade could be a reasonable time window to examine significant improvements in his face perception. However, since we cannot be sure that from this point in time LG will follow a typical maturation process, it is hard to predict whether such a significant change will indeed take place and what its effective size will then be. fMRI examinations of LG's visual system (Gilaie-Dotan *et al*., 2009) suggest that LG's visual stream has not successively developed and that his intermediate visual areas may remain not fully developed. Intermediate visual areas that are associated with fine resolution processing (Amedi, Malach, Hendler, Peled & Zohary, 2001; Grill-Spector, Kushnir, Edelman, Avidan, Itzchak & Malach, 1999; Lerner *et al*., 2001; Lerner, Hendler & Malach, 2002) are not normally developed in amblyopia (Lerner, Hendler, Malach, Harel, Leiba, Stolovitch & Pianka, 2006; Lerner, Pianka, Azmon, Leiba, Stolovitch, Loewenstein, Harel, Hendler & Malach, 2003). Thus, it is possible that the development of the visual stream might not be hierarchical and typically ‘leaves a gap’ such that higher-level perceptual areas develop before intermediate areas. Future examination of LG's high-level visual perceptual functions might assist in resolving this issue.

In summary, we found that in a young adult with a number of significantly underdeveloped visual functions relative to adult typical vision, visual training based on a perceptual learning procedure was effective in significantly improving trained and untrained basic visual functions. Moreover, it resulted in further improvements in untrained mid-level vision and indications for a mild progress in higher perceptual abilities as well. Further improvements could be expected with time, based on our experience with trained amblyopes (Polat *et al*., 2004). Since our results indicate that non-invasive treatment administered years after the ‘critical period’ is effective in improving vision, and since this training procedure is also effective in improving visual functions in a range of visual conditions (e.g. child and adult amblyopia, myopia), we anticipate that administering non-invasive visual treatments to individuals with underdeveloped or subnormal vision might prove useful and generalize to untrained functions, even when applied during adolescence or adulthood.
